# Clinical Significance of Patient Disease Awareness in Atrial Fibrillation: Risk Profiles and Post‐Ablation Outcomes

**DOI:** 10.1002/joa3.70341

**Published:** 2026-04-19

**Authors:** Masanaru Sawada, Naoto Otsuka, Koichi Nagashima, Ryuta Watanabe, Yuji Wakamatsu, Satoshi Hayashida, Shu Hirata, Moyuru Hirata, Sayaka Kurokawa, Yasuo Okumura

**Affiliations:** ^1^ Division of Cardiology, Department of Medicine Nihon University School of Medicine Tokyo Japan

**Keywords:** atrial fibrillation, catheter ablation, disease awareness, patient education, recurrence

## Abstract

**Background:**

The clinical relevance of patient disease awareness on risk factor control and clinical outcomes after atrial fibrillation (AF) ablation remains incompletely understood.

**Methods:**

We conducted a prospective cohort study enrolling 133 patients undergoing an initial AF ablation. Disease awareness was assessed using the Jessa Atrial Fibrillation Knowledge Questionnaire (JAKQ) —a 16‐item instrument comprising 8 questions on general AF knowledge and 8 questions on oral anticoagulant therapy—administered before and 1 year after ablation. We divided them into the poor disease awareness group and good disease awareness group according to the median value (75%) of the JAKQ score about AF in general and compared the baseline patient characteristics and the 1‐year changes in the JAKQ score, blood pressure, laboratory data, echocardiographic parameters, and AF/atrial tachycardia (AT) recurrence rate between the groups.

**Results:**

Forty‐two (31.6%) patients were classified as having a poor disease awareness (< 75% of the JAKQ score about AF in general), which was associated with hypertension, diabetes, dyslipidemia, and greater left atrial volume (LAV). These trends in the poor disease awareness group remained unchanged 1 year after the ablation. During the 25.3 [15.7–34.9] month follow‐up, the AF/AT recurrence rate was significantly higher in the poor disease awareness than good disease awareness group (23.8% vs. 7.7%; *p* = 0.003 by the log‐rank test).

**Conclusions:**

Poor disease awareness was linked to lifestyle‐related diseases, greater LAV before and even 1 year after the ablation, making it a potential surrogate marker for AF/AT recurrence. These findings highlight the clinical significance of disease awareness for AF.

## Graphical abstract

Poor disease awareness in atrial fibrillation was associated with lifestyle‐related comorbidities, greater left atrial volume, and increased post‐ablation atrial tachyarrhythmia recurrence. Disease awareness may serve as a surrogate marker influencing clinical outcomes and reinforce the importance of structured patient education.

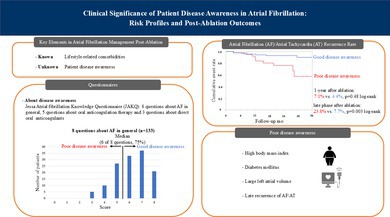

## 1 Introduction

Atrial fibrillation (AF) is the most common arrhythmia in adults and increases the risk of stroke. It is well‐known that AF is closely related to lifestyle‐related comorbidities such as hypertension, diabetes mellitus (DM), etc. [1], but is also associated with an increased risk of other cardiovascular events and all‐cause death [2].

Over the past decade, the use of oral anticoagulants became an established therapy to reduce strokes and stroke‐related deaths, and catheter ablation (pulmonary vein isolation [PVI]) has also been becoming an attractive therapy with the potential to prevent AF‐related clinical events including strokes, heart failure, worsening renal function, and dementia [3, 4, 5]. Even after AF ablation, an integrated and holistic approach for the management of AF is also important to prevent not only AF recurrence but also further clinical adverse events [6]. We hypothesized that the patient disease awareness regarding AF itself, AF‐related events, and its treatment, may also be one of the important elements to manage cardiovascular risk factors and prevent future clinical events because there has been a large gap reported between the patient disease awareness and AF treatment [7]. A valid AF‐specific knowledge questionnaire called the Jessa Atrial fibrillation Knowledge Questionnaire (JAKQ) is known to be used in routine practice to assess the patients' insight into their condition [8]. Nevertheless, there are no data on the relationship between the disease awareness and the time course changes in the comorbidities, and AF/atrial tachycardia (AT) recurrence after ablation. Therefore, we aimed to investigate the clinical effect of the patient disease awareness on the cardiovascular risk factors for the management after AF ablation.

## 2 Methods

### 2.1 Study Design

**FIGURE 1:**
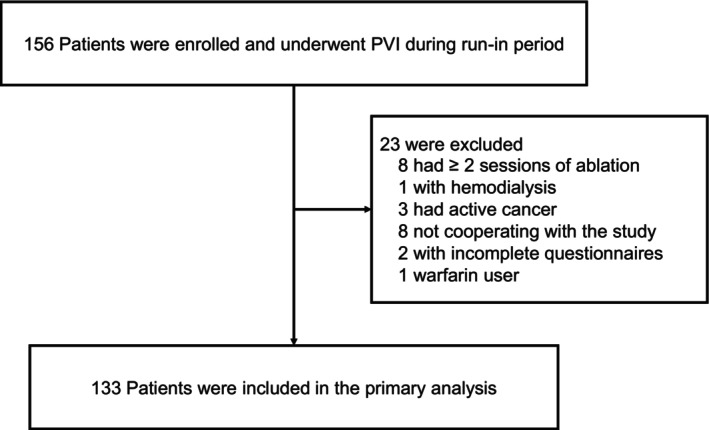
The patient flow of this study.

This study was a single‐center prospective observational study. The study participants included 156 consecutive patients who had undergone catheter ablation of paroxysmal AF (PAF; defined as AF returning to sinus rhythm within 7 days) and persistent AF (PerAF; defined as AF lasting ≥ 7 days) at Nihon University Itabashi Hospital between July 2019 and March 2020. The inclusion criteria were (1) patients who had initially undergone ablation of PAF or PerAF and (2) those who had been given questionnaires on their disease awareness before and 1 year after ablation. Exclusion criteria were (1) patients who had undergone ≥ 2 sessions of ablation, (2) hemodialysis patients, (3) active cancer patients, (4) patients not cooperating with the study, (5) patients with incomplete questionnaires, and (6) warfarin users after ablation. Of a total of 156 patients, we excluded 8 who had ≥ 2 sessions of ablation, 1 hemodialysis patient, 3 who had active cancer, 8 not cooperating with the study, 2 with incomplete questionnaires, and 1 warfarin user. As a result, 133 patients (86 men, 47 women; aged 65 ± 11 years) were enrolled in this study for the final analysis (Figure 1).

This study was conducted in accordance with the Declaration of Helsinki and the Ethical Guidelines for Medical and Health Research Involving Human Subjects in Japan. All participants provided written informed consent and could withdraw their consent at any time. This study protocol was approved by the Institutional Review Board (IRB) of Nihon University Itabashi Hospital, Clinical Research Judging Committee.

The Jessa Atrial Fibrillation Knowledge Questionnaire (JAKQ) was used under license from Hasselt University and Jessa Hospital, Belgium. The development and validation of the JAKQ has been previously described (8).

### 2.2 Clinical Data Collection

The patient information including the age, male sex, height, and body weight were collected before the ablation. The systolic and diastolic blood pressure (SBP and DBP) at baseline was collected in the morning during admission before the ablation. The blood pressure 1‐year post‐ablation was obtained from the average weekly home BP recordings. Laboratory data included the hemoglobin A1c (HbA1c), triglycerides (TG), total cholesterol, high‐density lipoprotein cholesterol, and low‐density lipoprotein cholesterol. The echocardiographic parameters included the left ventricular ejection fraction (LVEF), left atrial diameter (LAD), LA volume (LAV), E/A, and E/e’. The medication information including that of the antiarrhythmic drugs (AADs) and anticoagulants before and 1 year after ablation was collected.

### 2.3 Questionnaire

Prior to ablation, all patients participated in a standardized educational program consisting of an approximately 10‐min educational video followed by a face‐to‐face explanation lasting approximately 30 min. The educational content covered AF etiology, the cardiac conduction system and anatomy, the pathophysiology of AF, procedural aspects of catheter ablation, expected success rates, potential complications and their incidence, peri‐procedural and post‐discharge lifestyle guidance, and the use of direct oral anticoagulants (OACs) and AADs as part of the informed consent process. These sessions were delivered by four outpatient attending physicians using the same educational materials and a structured framework to ensure consistency. At 3–8 weeks following this educational intervention, all patients completed a questionnaire assessing disease awareness within 1 week before ablation and again 1 year after ablation. The questionnaires were based on the JAKQ for disease awareness [8]. The JAKQ questionnaire on disease awareness included 16 questions consisting of 8 questions about AF in general and 8 questions about OAC therapy including direct oral anticoagulant (DOAC) questions. The JAKQ consisted of 3 choices for each question with only one correct answer. For each question, there was an “I do not know” option to avoid guessing. A correct answer was scored as 1 point and an incorrect or “I do not know” answer was scored as 0 points. The total score was calculated from the completed questions and separately displayed as the percentage for the questions about AF in general and those about OAC therapy (Table S1). The questionnaires were collected by the doctors. Data verification was performed subsequently by another person. In this study, the JAKQ about AF in general was used to investigate the association between the disease awareness and AF/AT recurrence after ablation. That was because the 8 DOAC questions might have affected the clinical events including strokes and bleeding but might not have been directly associated with the post‐ablation AF/AT recurrence. The median percentage of the JAKQ about AF in general was 75%. Therefore, we defined ≥ 75% as good disease awareness and < 75% as poor disease awareness. This cut‐off was derived from the median score of the study cohort and is consistent with prior studies using the JAKQ, including the SAGE‐AF study [9].

### 2.4 Ablation Methods

For all patients, AADs were discontinued for at least 5 half‐lives prior to the ablation procedure, and oral anticoagulants were generally discontinued on the day of ablation. Conscious sedation was achieved with dexmedetomidine, propofol, and fentanyl. Vascular access was obtained, a single transseptal puncture guided by intra‐cardiac ultrasound was performed, and intravenous heparin was administered to maintain an activated clotting time of > 300 s. Three‐dimensional maps of the LA and 4 PVs were created with CARTO 3 (Biosense Webster) or the NavX system (Abbott Laboratories, Abbott Park, IL). An extensive encircling PVI was guided by a circular mapping catheter or multiple‐electrode catheter and a 3D mapping system. The ablation catheter was an irrigated‐tip contact force (CF) sensing catheter (SmartTouch SF [Biosense Webster Inc.] or TactiCath [St. Jude Medical, St. Paul, MN]) with a target CF of 10–15 g, and the ablation settings were based on an ablation index of 450 or lesion index of 5.5 for anterior sites and an AI of 350–400 or LSI of 4.5–5.0 for posterior sites with a power of 35 W. Regarding the balloon ablation technologies, an Arctic Front Advance cryoballoon (Medtronic, Minneapolis, MN, USA), the SATAKE HotBalloon ablation system (Toray Industries Inc., Tokyo, Japan), and a visually guided laser balloon (HeartLight Endoscopic Ablation System; CardioFocus, Marlborough, MA, USA) were used as described previously [10, 11, 12]. Any touch‐up ablation required for dormant conduction or residual PV potentials was performed with a standard irrigated‐tip catheter. In all patients except for those that fulfilled adenosine triphosphate contraindications, adenosine triphosphate was injected intravenously after the PVI to provoke any dormant PV conduction. If acute PV reconnections or dormant PV conduction was evident, touch‐up ablation was performed. In patients with long‐standing AF, a box isolation was performed as appropriate. A tricuspid valve isthmus ablation and superior vena cava isolation were also performed when necessary.

### 2.5 Post‐Ablation Follow‐Up and Endpoints

On the day after the ablation procedure, all AADs previously prescribed were resumed at the individual operator's discretion. Routine follow‐up was performed at the hospitals' respective outpatient clinics, where physical examinations and 12‐lead electrocardiography were performed at 2 weeks, 1 month, and every 3 months thereafter. Twenty‐four‐hour Holter recordings were obtained at 3, 6, and 12 months and every 1 year thereafter. Any symptomatic or documented atrial arrhythmias of ≥ 30 s after a 3‐month blanking period were taken as a recurrence of the AF/AT. During the follow‐up, all patients received a face‐to‐face standard interview regarding the patient's AF‐related symptoms and conditions, which were generally conducted at the outpatient clinic.

### 2.6 Study Endpoint

The primary endpoint was the annual AF/AT recurrence rate after ablation between a poor and a good disease awareness. The secondary endpoint was the late‐phase AF/AT recurrence rate at 2 years post‐ablation between the two groups. The tertiary endpoint was the time‐course change in the parameters such as the patient background, laboratory data, and transthoracic echocardiography between the two groups.

### 2.7 Statistical Analysis

Categorical variables are presented as the number and percentage of patients, and the differences in those variables between the patients in the good disease awareness group and in the poor disease awareness group were analyzed by a chi‐square or Fisher's exact test. Continuous variables are presented as the mean ± SD or median (and interquartile range) values, and the between‐group differences in those variables were analyzed by a Student's *t*‐test or Wilcoxon rank sum test. Serial changes in variables were evaluated by paired *t*‐test or Wilcoxon signed‐rank test. Multivariable logistic regression analysis was performed using forced entry methods for four explanatory variables potentially related to poor disease awareness: age, sex, DM, and asymptomatic AF. The cumulative event rate was estimated by the Kaplan–Meier method, and the differences were analyzed by a log‐rank test. Cox proportional hazards modeling was performed to identify the significant factors for the AF/AT recurrence. Factors shown to be significant by the univariable analysis were entered into the multivariable Cox model. All statistical analyses were performed with JMP 13.2.1 software (SAS Institute, Cary, NC), and a *p* < 0.05 was considered significant.

## 3 Results

### 3.1 Patient Characteristics

**FIGURE 2:**
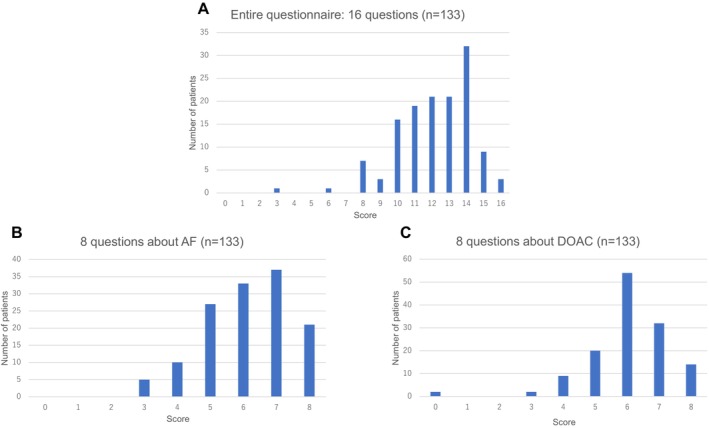
Frequency distribution of the scores on the JAKQ. (A) The scores in the patients who completed the entire questionnaire of 16 questions (*n* = 133). (B) Scores for the 8 questions about AF in general (*n* = 133). (C) Scores for the 8 questions about DOAC therapy (*n* = 133). AF, atrial fibrillation; DOAC, direct oral anticoagulation; JAKQ, jessa atrial fibrillation knowledge questionnaire.

The patient characteristics are shown for the total study patients and per study group in Table 1. The total male: female ratio was 86:47 and the mean age was 65 ± 11 years. AF was paroxysmal (lasting < 7 days) in 77 (57.9%) patients, persistent (lasting ≥ 7 days to 12 months) in 56 (42.1%), and long‐lasting persistent (lasting ≥ 12 months) in none. Radiofrequency ablation was performed in 91 (68.4%) patients and balloon technology was used in 42 (31.6%), including CBA in 31 (23.3%), HBA in 2 (1.5%), and LBA in 9 (6.8%). The distribution of the JAKQ in these study participants is presented in Figure 2. The mean score on the JAKQ was 76.0% ± 13.5%, including 76.6% ± 16.5% for 8 questions about AF in general and 75.4% ± 1.4% for 8 DOAC questions. Of a total of 133 patients, 42 (31.6%) were classified as having a poor disease awareness with < 75% of the JAKQ score about AF in general, while 91 (68.4%) were classified as having a good disease awareness with ≥ 75%. There were no differences in the age, male sex, height, body weight, SBP, DBP, smoking, alcohol consumption, and type of AF between the poor and good disease awareness groups, but the body mass index (BMI) tended to be higher in the poor disease awareness group than in the good disease awareness group (25.7 ± 4.3 vs. 24.4 ± 4.0 kg/m^2^; *p* = 0.06). Asymptomatic AF was significantly more prevalent in the poor disease awareness group (45.2% vs. 24.2%; *p* = 0.015). Hypertension was more likely to be observed (61.9% vs. 44.0%; *p* = 0.05) and DM and dyslipidemia were significantly more prevalent in the poor disease awareness group than in the good disease awareness group (DM: 31.0% vs. 11.0%; *p* = 0.005; dyslipidemia: 45.2% vs. 24.2%; *p* = 0.015). There were no differences in prior strokes and vascular disease between the two groups. Regarding the laboratory data, there were no differences in any of the parameters between the two groups. As for the echocardiographic variables, the LAD and LAV were greater in the poor disease awareness group than in the good disease awareness group (LAD 42.1 ± 7.0 mm vs. 39.8 ± 6.1 mm; *p* = 0.05; LAV 56.8 ± 23.0 mL vs. 49.3 ± 19.0 mL; *p* = 0.049). There were no differences in the LVEF, E/A, and E/e’ between the two groups. Multivariable analysis revealed that DM and asymptomatic AF were still associated with poor disease awareness (Odds ratios 3.36 [95% confidence interval (CI) 1.27–8.94]; *p* = 0.015, and 2.33 [95% CI 1.04–5.22]; *p* = 0.039, respectively).

**TABLE 1:** Baseline patient clinical, laboratory data, and echocardiographic characteristics, per study group.

	Poor disease awareness (*n* = 42)	Good disease awareness (*n* = 91)	*p*
Clinical characteristics			
Age (years)	65.6 ± 8.8	64.4 ± 12.1	0.57
Male sex	29 (69.1%)	60 (65.9%)	0.72
Height (cm)	165.3 ± 10.0	164.5 ± 9.8	0.66
Body weight (kg)	70.6 ± 14.8	66.2 ± 14.0	0.11
BMI (kg/m^2^)	25.7 ± 4.3	24.4 ± 3.95	0.08
SBP (mmHg)	129.6 ± 15.1	126.9 ± 13.9	0.32
DBP (mmHg)	78.5 ± 11.8	78.8 ± 11.2	0.87
Smoking	29 (71.4%)	54 (58.1%)	0.23
Alcohol	19 (45.2%)	47 (51.7%)	0.49
AF type			
PAF	22 (52.4%)	55 (60.4%)	0.38
PerAF	20 (47.6%)	36 (39.6%)	0.38
Asymptomatic	19 (45.2%)	22 (24.2%)	0.015
Comorbidities			
Heart failure	2 (4.76%)	12 (12.9%)	0.15
Hypertension	26 (61.9%)	40 (44.0%)	0.05
Diabetes mellitus	13 (31.0%)	10 (11.0%)	0.005
Dyslipidemia	19 (45.2%)	23 (24.2%)	0.015
Prior stroke	2 (4.8%)	6 (6.6%)	0.68
Vascular disease	1 (2.4%)	1 (1.1%)	0.57
CHADS_2_ score	1.2 ± 0.9	1.1 ± 1.1	0.48
CHA_2_DS_2_‐VASc score	2.1 ± 1.4	2.0 ± 1.5	0.70
Antiarrhythmic drug use at baseline	12 (28.6%)	52 (57.1%)	0.002
Class I	8 (19.1%)	32 (35.2%)	0.06
Class III	0	1 (1.1%)	0.50
Class IV	5 (11.9%)	23 (25.3%)	0.08
Laboratory data			
HbA1c	6.1 ± 0.7	6.0 ± 0.6	0.13
TG (mg/dL)	135.4 ± 75.9	122.6 ± 68.2	0.34
TC (mg/dL)	200.8 ± 28.8	199.1 ± 36.1	0.79
HDL‐C (mg/dL)	56.9 ± 12.3	58.2 ± 15.1	0.62
LDL‐C (mg/dL)	117.4 ± 27.0	116.2 ± 30.7	0.84
Echocardiographic variables			
LVEF (%)	65.6 ± 8.5	65.5 ± 7.6	0.98
LAD (mm)	42.1 ± 7.0	39.8 ± 6.1	0.05
LAV (mL)	56.8 ± 23.0	49.3 ± 19.0	0.049
E/A	1.3 ± 0.8	1.2 ± 0.5	0.62
E/e’	10.7 ± 4.3	10.2 ± 3.9	0.50

*Note:* Mean ± SD or median (25th, 75th percentile) values or number (%) of patients are shown.

Abbreviations: BMI, body mass index; DBP, diastolic blood pressure; HDL‐C, high density lipoprotein cholesterol; LAD, left atrial diameter; LDL, low density lipoprotein cholesterol; LVEF, left ventricular ejection fraction; SBP, systolic blood pressure; TC, total cholesterol; TG, triglyceride.

### 3.2 The Serial Changes in the Parameters Potentially Related to AF Progression Before and 1 Year After Ablation

The changes in various parameters before and 1 year after ablation between a poor and good disease awareness are provided in Table 2. In both the poor and good disease awareness groups, the BMI and HbA1c tended to decrease. The SBP was significantly elevated after ablation in the poor disease awareness group (from 129.6 ± 15.1 at baseline to 135.3 ± 15.5 mmHg after ablation; *p* = 0.042), whereas it was not in the good disease awareness group (from 127.0 ± 13.9 to 129.8 ± 13.6 mmHg, *p* = 0.09). The HbA1c tended to decrease after ablation in the poor disease awareness group, but it decreased significantly in the good disease awareness group (from 6.1% ± 0.7% to 5.9% ± 0.7%; *p* = 0.06 and from 6.0 ± 0.6 to 5.8% ± 0.5%; *p* < 0.001). The TG levels were significantly increased in the poor awareness group, while they did not change in the good awareness group. As for echocardiographic parameters, both the poor and good disease awareness groups had a significant reduction in the LAD and LAV (*p* < 0.001 for all). In the poor disease awareness group, the mean score for disease awareness about AF in general improved from 56.5 ± 8.8 to 68.8% ± 13.3% (*p* < 0.001), while no significant change was observed in the good disease awareness group (from 85.9 ± 9.5 to 85.4% ± 9.4%; *p* = 0.62). In the poor disease awareness group, the mean score for disease awareness about DOACs decreased from 68.2 ± 18.6 to 66.4% ± 14.7% (*p* = 0.45), while an improvement was observed in the good disease awareness group (from 78.7 ± 14.6 to 80.1% ± 10.9%; *p* = 0.27), but both differences were not significant. Despite the significant improvement in the disease awareness in the poor disease awareness group, those scores remained significantly lower than in the good disease awareness group (*p* < 0.001).

**TABLE 2:** The changes in the various parameters before and 1‐year after ablation between the poor and good disease awareness groups.

	Disease awareness					
	Poor			Good		
	Baseline	Post	*p*	Baseline	Post	*p*
BMI (kg/m^2^)	25.7 ± 4.3	25.4 ± 4.3	0.07	24.4 ± 4.0	24.2 ± 4.0	0.06
SBP (mmHg)	129.6 ± 15.1	135.3 ± 15.5	0.042	127.0 ± 13.9	129.8 ± 13.6	0.09
DBP (mmHg)	78.5 ± 11.8	80.5 ± 10.7	0.28	78.8 ± 11.2	79.7 ± 12.5	0.48
HbA1c	6.1 ± 0.7	5.9 ± 0.7	0.06	6.0 ± 0.6	5.8 ± 0.5	< 0.001
TG (mg/dL)	127.7 ± 75.9	152.5 ± 79.8	0.02	122.3 ± 68.5	132.0 ± 86.7	0.28
TC (mg/dL)	197.5 ± 28.8	200.4 ± 31.1	0.55	199.9 ± 36.1	200.3 ± 35.4	0.92
HDL‐C (mg/dL)	57.9 ± 12.3	57.7 ± 14.5	0.91	58.4 ± 15.3	58.6 ± 15.7	0.83
LDL‐C (mg/dL)	114.5 ± 27.0	112.0 ± 25.6	0.49	116.9 ± 30.7	114.0 ± 27.0	0.35
LVEF (%)	66.0 ± 8.5	69.2 ± 4.8	0.025	65.8 ± 7.6	68.2 ± 6.8	0.004
LAD (mm)	42.3 ± 7.0	38.8 ± 7.4	< 0.001	39.8 ± 6.1	37.4 ± 5.5	< 0.001
LAV (mL)	57.5 ± 23.0	46.4 ± 20.3	< 0.001	48.9 ± 19.0	41.4 ± 15.2	< 0.001
E/A	1.3 ± 0.8	1.1 ± 0.5	0.12	1.2 ± 0.5	1.2 ± 0.5	0.98
E/e’	11.0 ± 4.3	9.8 ± 3.6	0.031	10.4 ± 3.6	9.7 ± 3.3	0.08
Disease awareness about AF in general (%)	56.5 ± 8.8	68.8 ± 13.3	< 0.001	85.9 ± 9.5	85.4 ± 9.4	0.62
Disease awareness about DOAC therapy (%)	68.2 ± 18.6	66.4 ± 14.7	0.45	78.7 ± 14.6	80.1 ± 10.9	0.27

*Note:* Mean ± SD or median are shown. The abbreviations are as shown in Table 1. “Disease awareness about AF in general” and “Disease awareness about DOAC therapy” represent scores derived from the respective sub‐sections of the Jessa Atrial Fibrillation Knowledge Questionnaire (JAKQ). Each value indicates the percentage of correctly answered questions within the corresponding section.

### 3.3 AF/AT Recurrence Rate at 1 Year and Thereafter Between the Patients With a Poor Disease Awareness and Good Disease Awareness

**FIGURE 3:**
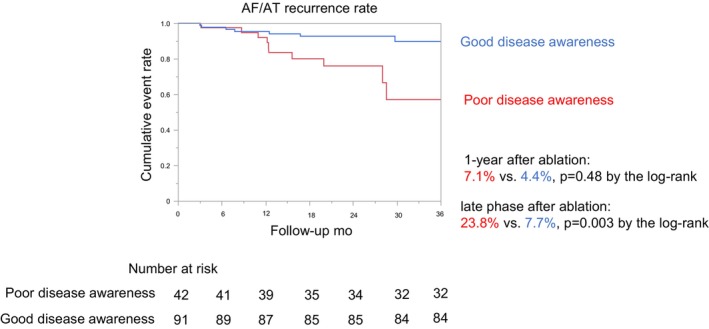
Kaplan–Meier curve of the AF/AT recurrence rate at 1‐year and during the late phase after ablation. The median (25th, 75th percentile) length of the patient follow‐up was 25.3 (15.7, 34.9) months. AF, atrial fibrillation; AT, atrial tachycardia.

The Kaplan–Meier freedom rate from AF/AT recurrence showing a comparison of the endpoints in the two groups is provided in Figure 3. The use of AADs at baseline was significantly lower (28.6% vs. 57.1%; *p* = 0.002), but that at 1 year and 2 years after the ablation tended to be higher in poor disease awareness group than good disease awareness group (1 year after ablation 59.5% vs. 41.8%; *p* = 0.06: 2 year after ablation 47.6% vs. 38.5%; *p* = 0.32). There was no significant difference in the annual AF/AT recurrence rate between the poor disease awareness and good disease awareness groups (7.1% vs. 4.4%; *p* = 0.48 by the log‐rank test). Nevertheless, the AF/AT recurrence rate in the late phase (median 25.3 [15.7–34.9] months) increased significantly more in patients with a poor disease awareness than in those without (23.8% vs. 7.7%; *p* = 0.003 by the log‐rank test). Consistent with this finding, a 1‐year landmark analysis demonstrated that recurrence beyond 1 year occurred in 7 of 33 patients (21.2%) in the poor‐awareness group and in 4 of 77 patients (5.2%) in the good‐awareness group (*p* = 0.003 by the log‐rank test). The results of the Cox regression analysis evaluating the relative risk of an AF/AT recurrence are summarized in Table 3. In the univariable Cox proportional hazards model analysis, the BMI, LAV, and DM were also associated with AF/AT recurrence (BMI, hazard ratio [HR] 1.15 [95% CI 1.04–1.26] per 1 kg/m^2^ increase; *p* = 0.005: LAV, HR 1.03 [95% CI 1.01–1.05] per 1 mL increase; *p* = 0.002: DM, HR 3.22 [95% CI 1.19–8.73]; *p* = 0.022). A multivariable Cox proportional hazards analysis was performed using three models including (1) the disease awareness and BMI, (2) the disease awareness and LAV, and (3) the disease awareness and DM, but in all models, a poor disease awareness remained to be significantly associated with AF/AT recurrence (model 1: poor disease awareness, HR 3.51 [95% CI 1.30–9.45]; *p* = 0.013 and BMI, HR 1.14 [95% CI 1.02–1.25] per 1 kg/m^2^ increase; *p* = 0.013: model 2: poor disease awareness, HR 3.00 [95% CI 1.08–8.35]; *p* = 0.035 and LAV, HR 1.02 [95% CI 1.00–1.04] per 1 mL increase; *p* = 0.019: model 3: poor disease awareness, HR 3.48 [95% CI:1.28–9.44]; *p* = 0.014 and DM, HR 2.51 [95% CI 0.91–6.97], *p* = 0.08). In addition, in a parsimonious multivariable Cox proportional hazards model including disease awareness and CHA_2_DS_2_‐VASc score as a representative indicator of baseline clinical risk, poor disease awareness was associated with a higher risk of AF/AT recurrence (HR 3.99 [95% CI: 1.50–10.6]; *p* = 0.006), whereas the CHA_2_DS_2_‐VASc score was not significantly associated with recurrence (HR 1.07 [95% CI: 0.70–1.63]; *p* = 0.74).

**TABLE 3:** Univariate and multivariate cox hazard models for the relationship between AF/AT recurrence and the various parameters.

	HR (95% CI)	*p*
Clinical characteristics		
Age (+1 year)	1.00 (0.96–1.05)	0.98
Male sex	0.57 (0.19–1.76)	0.33
Height (+1 cm)	1.00 (0.95–1.05)	0.94
Body weight (+1 kg)	1.04 (1.01–1.07)	0.017
BMI (+1 kg/m^2^)	1.15 (1.04–1.26)	0.005
SBP (+1 mmHg)	1.00 (0.97–1.04)	0.90
DBP (+1 mmHg)	1.00 (0.96–1.05)	0.90
Smoking	1.68 (0.59–4.77)	0.33
Alcohol	0.71 (0.27–1.87)	0.49
AF type		
PAF	0.41 (0.15–1.11)	0.08
PerAF	2.44 (0.90–6.63)	0.08
Asymptomatic	2.26 (0.87–5.85)	0.09
Comorbidities		
Heart failure	1.03 (0.23–4.53)	0.97
Hypertension	2.44 (0.86–6.92)	0.095
Diabetes mellitus	3.22 (1.19–8.73)	0.022
Dyslipidemia	2.46 (0.94–6.41)	0.07
Prior stroke	0.76 (0.10–5.7)	0.79
CHADS_2_ score	1.27 (0.83–1.82)	0.23
CHA_2_DS_2_‐VASc score	1.08 (0.79–1.47)	0.61
Antiarrhythmic drug use at baseline		
Class I	1.21 (0.45–3.28)	0.70
Class III	—	—
Class IV	0.19 (0.02–1.43)	0.11
Laboratory data		
HbA1c (+1%)	1.79 (0.88–3.20)	0.07
TG (+1 mg/dL)	1.00 (0.99–1.00)	0.42
TC (+1 mg/dL)	1.00 (0.99–1.02)	0.81
HDL‐C (+1 mg/dL)	0.99 (0.96–1.03)	0.66
LDL‐C (+1 mg/dL)	1.01 (0.99–1.03)	0.35
Echocardiographic variables		
LVEF (+1%)	0.96 (0.92–1.02)	0.11
LAD (+1 mm)	1.07 (0.99–1.14)	0.08
LAV (+1 mL)	1.03 (1.01–1.05)	0.002
E/A (+1)	0.53 (0.07–1.12)	0.48
E/e’ (+1)	0.96 (0.85–1.09)	0.56
Questionnaire		
Poor disease awareness < 75%	3.99 (1.50–10.6)	0.006

*Note:* Mean ± SD or median are shown. The abbreviations are shown in Table 1.

## 4 Discussion

This study had three major findings: (1) a poor disease awareness was associated with a higher BMI, higher prevalence of comorbidities, asymptomatic AF and greater LAV, (2) a poor disease awareness can be a surrogate marker for recurrence of AF/AT after ablation even after multivariable adjustment for significant variables such as the BMI, LAV, or DM, and (3) regardless of a poor disease awareness or good disease awareness, most parameters related to AF progression improved, but an improvement in the SBP, HbA1c, and TG was less in those with a poor disease awareness. Despite the significant improvement in disease awareness, its score remained significantly lower in those with a poor disease awareness.

### 4.1 Clinical Significance of a Poor Disease Awareness

This study disclosed the relatively high score on the JAKQ in patients who planned to undergo AF ablation. The mean score on the JAKQ was 76.0% ± 13.5%, including 76.6% ± 16.5% for 8 questions about AF in general and 75.4% ± 1.4% for 8 questions about OAC therapy with DOACs, which was higher than that in those who did not have any education as reported previously [8]. In the original study, the mean score on the JAKQ was 55.8% ± 18.6%, including 51.6% for 8 questions about AF in general and 61.9% for 8 questions about OAC therapy with DOACs. In their study, as compared to the initial score on the JAKQ, 20 hospitalized AF patients scored significantly better about 2 days after they had received an individualized education (60.9% ± 16.6% vs. 78.8% ± 14.8%; *p* = 0.001), and a longer time span of 1 month after the initial completion of the JAKQ followed by targeted education also improved the scores in a different population of 20 AF patients (61.6% ± 14.5% vs. 76.9% ± 13.8%; *p* = 0.001). A randomized controlled trial also showed a significant improvement in the JAKQ score by a targeted educational session (62.5% to 87.5% 1 year) as compared to standard care (56.3% to 62.5% 1 year) [13]. Our patients received an educational video on AF and face‐to‐face education on AF ablation, and therefore, a better score was observed than in the prior studies [8, 13, 14, 15]. We also characterized the patients who were diagnosed with a poor disease awareness assessed by the JAKQ about AF in general. Patients with a poor disease awareness tended to have a greater BMI, and significantly higher prevalence of hypertension, DM, and a larger LAD and LAV. This study disclosed novel insights into understanding the changes in lifestyle diseases driven by routine follow‐up after ablation. A previous study showed that among patients with intensive risk factor management, the most marked improvements are seen with respect to hypertension and glycemic control, despite improvements across the board regarding lifestyle‐related diseases [16]. Reverse atrial remodeling in response to weight loss optimizes the results following ablation [17]. We did not intervene with any specific education during the follow‐up, but the disease awareness scores improved, and the BMI, HbA1c, LAD, and LAV all became reduced 1‐year post‐ablation, regardless of a poor or good disease awareness. That suggested an intervention by ablation followed by routine post‐ablation follow‐up may also provide a favorable effect on the lifestyle disease to some extent. However, only the poor disease awareness group had an elevated TG level at 1 year after ablation. The SBP increased significantly at 1 year after ablation in both the good and poor disease awareness groups, possibly due to sinus restoration after ablation. Nonetheless, its extent was significantly greater in the poor disease awareness group. The serial improvement in the HbA1c was statistically lesser in the poor disease awareness group. As a result, those scores remained lower and the BMI, HbA1c, and LAD larger at 1 year after ablation in the poor disease awareness group. Those findings not only highlight the importance of a baseline poor disease awareness, but also it might be a hindering factor for modifiable lifestyle diseases during a routine follow‐up after ablation. The unfavorable risk factor profiles observed at 1 year are more likely to act as drivers of gradual structural and electrophysiological remodeling rather than as triggers of short‐term arrhythmia recurrence, which may explain the lack of association with early recurrence. Over time, however, the cumulative impact of suboptimal risk factor control may promote progressive atrial remodeling, ultimately manifesting as late‐phase AF/AT recurrence. Consistent with this interpretation, landmark analysis demonstrated that divergence in recurrence rates became evident only during later follow‐up, supporting a temporal latency between risk factor status at 1 year and clinically manifest arrhythmia recurrence. The reason why a poor disease awareness had lesser serial favorable effects on lifestyle diseases remains unclear. The poor disease awareness group might be associated with asymptomatic AF, which might also have acted to hinder their disease awareness and the importance of the management of lifestyle diseases. Our study demonstrated that a poor disease awareness potentially led to a late AF/AT recurrence. A poor disease awareness in this study had more lifestyle diseases including metabolic syndrome. It is also well‐known that obesity and the components of the metabolic syndrome including hypertension, dyslipidemia, and DM are associated with a larger LAD [17, 18, 19]. Chang and colleagues [19] reported that patients with metabolic syndrome had a larger LAD, shorter fractionated intervals, and higher dominant frequencies as compared to those without. Furthermore, patients with metabolic syndrome have been found to experience more frequent recurrent AF [18, 19]. It has been reported that asymptomatic AF is associated with more comorbidities, a high thromboembolic risk, and higher 1‐year mortality than symptomatic patients [20]. Another previous study using the JAKQ scores in anticoagulated patients with AF also showed that lower baseline JAKQ scores had a higher incidence of the composite of ischemic cerebrovascular events, major or non‐major clinically relevant bleeding, and death [14]. The study also reported that intensive risk factor management, resulting in improved anthro‐morphometric profiles, cardiac structure, and symptom scores, led to a higher freedom from arrhythmia recurrence [16]. Therefore, it was not clear whether the recurrence of AF/AT was due to poor disease awareness, poor serial changes in modifiable risk factors, or the original patient background as multiple confounders. However, since poor disease awareness remains an independent factor, it can at least serve as a surrogate marker to identify patients at high‐risk for AF/AT recurrence. In light of these findings, enhancing disease awareness and emphasizing the importance of anticoagulant therapy may be modifiable factors for reducing the risk of future AF/AT recurrences and related clinical events, particularly in patients with limited disease awareness.

### 4.2 Study Limitations

This study had several limitations that should be considered. First, this study was a single‐center observational study, so no causal relationships could be established. Accordingly, the observed left atrial remodeling should be interpreted as descriptive rather than as evidence of a direct effect of disease awareness. Educated AF ablation patients were included, so it cannot be generalized to all AF patients. To extend the applicability of our findings, additional research based on a multicenter study is warranted. Second, there is currently insufficient clinically established evidence to support using a cut‐off of below 75% of the JAKQ about AF in general as an indicator of the “poor disease awareness”. It's important to note that this value was based on the median percentage of the scale, as described in the method section. As such, this study serves as an initial pilot study to explore the clinical significance of this threshold on outcomes. Further research and increasing evidence will be required to substantiate its validity. Third, several baseline characteristics, including BMI and the prevalence of DM, differed significantly between the poor and good disease awareness groups. Although we attempted to mitigate confounding by constructing separate parsimonious Cox proportional hazards models adjusting for disease awareness and one additional covariate (BMI, LAV, or DM), residual confounding cannot be excluded. Moreover, the relatively small number of AF/AT recurrence events limited statistical power and constrained the complexity of multivariable modeling. Therefore, the observed associations should be interpreted with appropriate caution. In a parsimonious multivariable model including disease awareness and CHA_2_DS_2_‐VASc score, poor disease awareness remained independently associated with recurrence, whereas the CHA_2_DS_2_‐VASc score was not. This suggests that the association is not fully attributable to baseline clinical risk. Fourth, post‐ablation recurrence may have been underestimated because follow‐up relied on symptom‐driven visits, periodic 12‐lead electrocardiography, and scheduled 24‐h Holter monitoring, which may miss asymptomatic or short‐lasting arrhythmias, especially in patients with lower disease awareness. Although surveillance was uniform across groups, differential detection cannot be ruled out. Fifth, the use of AADs 1 year and 2 years after ablation might have influenced our results. Nonetheless, the effects of the higher success rate in those with a good disease awareness than in those with a poor disease awareness might have been small because the use of AADs at 1 year and 2 years after the ablation were even lower in good disease awareness group. Finally, variability in routine clinical practice may have introduced heterogeneity in both the assessment of disease awareness and post‐ablation follow‐up. Baseline JAKQ assessment was performed approximately 3–8 weeks after the educational intervention, and variation in this interval may have influenced knowledge retention. In addition, routine post‐ablation follow‐up, including standardized face‐to‐face interviews, was conducted at the discretion of the attending physicians. However, both the poor and good disease awareness groups were managed by the same four outpatient attending physicians, and all providers adhered to the same educational materials and a structured framework. This approach ensured consistency of educational content across patients and may have partially mitigated systematic bias between groups.

## 5 Conclusions

A poor disease awareness was associated with hypertension, DM, and a large LAD and LAV. Regardless of baseline disease awareness status, the LAD and LAV decreased 1 year after ablation, but patients with a poor disease awareness remained to have a larger LAD and LAV and a lower disease awareness than those with a good disease awareness. A poor disease awareness was also associated with AF/AT recurrences after ablation. These findings suggest that disease awareness could be an additive factor that influences clinical outcomes in the context of post‐ablation AF management, especially for patients with a poor understanding of AF.

## Author Contributions

Masanaru Sawada and Yasuo Okumura wrote the first draft of the protocol manuscript and carry the overall responsibility for the full study and the study protocol. Yasuo Okumura and Koichi Nagashima were substantial contributors to the study concept and design, manuscript drafting, and critical review of the manuscript and will contribute to the acquisition, analysis, and interpretation of the data. Masanaru Sawada, Naoto Otsuka, Koichi Nagashima, Ryuta Watanabe, Yuji Wakamatsu, Satoshi Hayashida, Moyuru Hirata, Shu Hirata, and Sayaka Kurokawa collected the data and conducted the study and have approved the final version of this manuscript. Yasuo Okumura gave us critical comments on the statistical methods and contributed to the analysis and interpretation of the data.

## Funding

This work is own‐funded.

## Disclosure

Approval of Research Protocol: The study was approved by the Institutional Review Board of Nihon University Itabashi Hospital, and an opt‐out system was used to obtain the patients' consent for the use of their clinical data for research purposes.

## Consent

Written informed consent was obtained from all participants.

## Conflicts of Interest

The authors declare the following competing interests. Yasuo Okumura reports honoraria from AstraZeneca and Johnson & Johnson/Biosense Webster; research funding from Medtronic Japan, MicroPort CRM Japan, and Bayer Healthcare; and an endowed chair from Abbott Medical Japan, Japan Lifeline, Medtronic Japan, Boston Scientific Japan K.K., and Biotronik Japan. He has also received lecture fees from Abbott Japan LLC, Bristol‐Myers Squibb, AstraZeneca K.K., Daiichi Sankyo Co. Ltd., Boston Scientific Japan K.K., Johnson & Johnson/Biosense Webster, Bayer Yakuhin Ltd., and Pfizer Japan Inc. K.N. received honoraria from Daiichi Sankyo Co. Ltd., Johnson & Johnson/Biosense Webster, Medtronic Japan, Boston Scientific Japan K.K., and Abbott Medical Japan. The other authors declare no conflicts of interest.

## Supporting information


**Table S1:** Items of the Jessa Atrial fibrillation Knowledge Questionnaire.

## Data Availability

The data that support the findings of this study are available on request from the corresponding author. The data are not publicly available due to privacy or ethical restrictions.
