# Supplementary material for: Clinical Significance of Patient Disease Awareness in Atrial Fibrillation: Risk Profiles and Post‐Ablation Outcomes
Source: J Arrhythm. 2026 Apr 19;42(2):e70341. doi: 10.1002/joa3.70341 (PMC13092948; doi:10.1002/joa3.70341)
Supplement: Supplementary file 1 — Table S1: Items of the Jessa Atrial fibrillation Knowledge Questionnaire. [file JOA3-42-e70341-s001.docx]

| Supplementary table. Items of the Jessa Atrial fibrillation Knowledge Questionnaire |
| --- |
| **8 questions about AF in general** |
| AF is a condition where the heart beats irregularly and often faster than normal |
| AF is not always accompanied by symptoms |
| Patients can detect AF by taking their pulse regularly |
| AF can cause blood clots which can lead to stroke (cerebral infarction) |
| Medication cannot prevent AF permanently, as the arrhythmia will increasingly occur with ageing, even when taking medication |
| An AF patient should not go to the general practitioner or emergency room each time he/she feels AF |
| Being overweight exacerbates AF |
| Blood thinners are often prescribed for patients with AF in order to prevent the development of blood clots in the heart, which can lead to stroke |
| **8 questions about OAC therapy including DOAC questions** |
| Patients with AF should always take their blood thinners, even if they do not feel AF |
| For patients taking NOAC, it is important to take their blood thinner at the same time every day |
| AF patients may only take painkillers based on paracetamol |
| When AF patients taking NOAC have forgotten to take their blood thinner, they can still take that dose, unless the time till the next dose is less than the time after the missed dose |
| Possible side effects of blood thinners are the occurrence of bleedings and longer bleeding times in case of injuries |
| NOAC blood thinners come with a card, which AF patients have to show to their general practitioner and specialist |
| When AF patients regularly have minor nose bleeds (that spontaneously cease), they should contact the general practitioner or specialist, while continuing to take their blood thinners |
| If an AF patient needs an operation, he/she should consult a doctor to discuss possible options |

Supplementary table
